# In Search of New Therapeutic Targets in Obesity Treatment: Sirtuins

**DOI:** 10.3390/ijms17040572

**Published:** 2016-04-19

**Authors:** Alina Kurylowicz

**Affiliations:** Department of Human Epigenetics, Mossakowski Medical Research Centre, Polish Academy of Sciences, 5 Pawinskiego Street, 02-106 Warsaw, Poland; akurylowicz@imdik.pan.pl; Tel.: +48-22-608-65-91; Fax: +48-22-608-64-10

**Keywords:** sirtuins, obesity, metabolism, genes expression, single nucleotide polymorphisms

## Abstract

Most of the available non-invasive medical therapies for obesity are non-efficient in a long-term evaluation; therefore there is a constant need for new methods of treatment. Research on calorie restriction has led to the discovery of sirtuins (silent information regulators, SIRTs), enzymes regulating different cellular pathways that may constitute potential targets in the treatment of obesity. This review paper presents the role of SIRTs in the regulation of glucose and lipid metabolism as well as in the differentiation of adipocytes. How disturbances of SIRTs’ expression and activity may lead to the development of obesity and related complications is discussed. A special emphasis is placed on polymorphisms in genes encoding SIRTs and their possible association with susceptibility to obesity and metabolic complications, as well as on data regarding altered expression of SIRTs in human obesity. Finally, the therapeutic potential of SIRTs-targeted strategies in the treatment of obesity and related disorders is discussed.

## 1. Introduction

Changes in the lifestyle and dietary habits over the last decades have resulted in an “outbreak” of a worldwide pandemic of obesity that shortens the lifespans of the affected individuals, and together with the associated metabolic complications, constitute a significant socioeconomic problem. Obesity, especially when associated with the accumulation of visceral adipose tissue, shortens lifespan indirectly by increasing the risk of developing many diseases, including hypertension, type 2 diabetes mellitus (T2DM), and hyperlipidemia—major components of metabolic syndrome. Apart from the strictly medical aspects, the growing prevalence of obesity is a significant socio-economic problem. It has been shown that obesity negatively affects personal and working relations and it is estimated that treatment of overweight and obese individuals’ costs, on average, 30%–40% more than health care for people of normal weight [1].

Major goals in the treatment of obesity include not only weight reduction, but also a reduction of obesity-related complications such as insulin-resistance, hyperlipidemia and cardiovascular diseases. The most commonly used method of treating obesity is calorie restriction (CR) combined with increased physical activity; however, long-term effects of dietary interventions are frequently disappointing. Apart from bariatric surgery, which is effective but also the most expensive, efficacy of other medical therapies is limited and therefore there is a constant need for novel, non-invasive methods for the treatment of obesity and related complications.

Paradoxically, in the research on the pathogenesis and consequences of obesity, a state of permanent over-nutrition, studies on CR have been particularly helpful. These studies identified sirtuins (silent information regulators, SIRTs) as important players in different cellular metabolic pathways and seem to be interesting therapeutic targets in the treatment of obesity and related complications.

## 2. Short Review of the Silent Information Regulators (SIRTs) System

The SIRTs comprise of a family of highly conserved regulatory proteins present in virtually all species. Originally, SIRTs were identified as class III histone deacetylases, nicotinamide adenine dinucleotide (NAD) dependent enzymes responsible for the removal of acetyl groups from lysine residues in proteins; however, some members of this family also act as mono-ADP-ribosyltransferases. Acetylation and deacetylation is an important mechanism of posttranslational modifications responsible for protein activation or inactivation and therefore, for the regulation of distinct cellular pathways. Indeed, the list of SIRTs targets in mammals includes, among others, those involved in the regulation of cell survival, apoptosis, inflammatory and stress responses, as well as lipid and glucose homeostasis [2].

In mammals, seven *SIRT* genes (*SIRTs*) have been identified that encode seven distinct SIRT enzymes of different structure, cellular localization, and tissue expression. Despite variable length and sequence, all *SIRTs* have a highly conserved catalytic core region consisting of approximately 275 amino acids, forming a Rossmann-fold domain (characteristic of NAD^+^/NADH binding proteins) and a zinc-binding domain connected by several loops [2]. Outside the catalytic core, SIRT enzymes possess variable N- and C-terminal regions that determine their enzymatic activities, binding partners and substrates, as well as subcellular localization [3]. SIRT1, SIRT6 and SIRT7 are predominantly found in the nucleus where via modifications of transcription factors, cofactors and histones they participate in the regulation of energy metabolism, stress and inflammatory responses, DNA repair (SIRT1 and SIRT6), and rDNA transcription (SIRT7) [4]. SIRT2 is present in the cytoplasm and primarily plays a role in cell cycle control [5]. SIRT3 is located in mitochondria and takes part in the regulation of metabolic enzymes (e.g., those involved in glycolysis, fatty acid (FA) oxidation, ketone body synthesis and amino acid catabolism), apoptosis and oxidative stress pathways. SIRT3 also exists as a nuclear full length form (FL-SIRT3) that is subsequently processed to the short mitochondrial form. Therefore, SIRT3 may regulate cellular metabolism both at the transcriptional and posttranscriptional level. SIRT4 is also localized in mitochondria and acts as ADP-ribosylase. Another mitochondrial sirtuin, SIRT5, has potent demalonylation and desuccinylation enzymatic activity and is involved in the regulation of amino acid catabolism [6]. However, the subcellular localization of SIRTs may depend on cell type and their molecular interactions, as it was shown in the case of SIRT1, SIRT2 and SIRT3, which can be found both in the nucleus and cytoplasm [4].

Expression of *SIRTs* was detected in various human tissues, including hypothalamus, liver, pancreatic islets, skeletal muscles and adipocytes [7,8,9,10]. In these tissues, via modification of histones, as well as transcription factors and co-regulators, *SIRTs* controlled expression of other genes, particularly those involved in response to stress. It was shown that SIRTs expression and activity of SIRT enzymes are highly sensitive to several environmental factors, including CR, exercise and cold exposure, as an adaptive mechanism in response to environmental stress [3]. Fluctuations in intracellular NAD^+^ levels in response to nutrient availability are believed to be a chief mediator in this phenomenon. When nutrients are plentiful, cellular metabolism relies mainly on glycolysis to produce energy, leading to generation of ATP and conversion of NAD^+^ to NADH. Low levels of NAD^+^ and high levels of NADH result in the inactivation of SIRTs enzymatic activity. In turn, CR (defined as a diet that supplies all essential nutrients, but its energetic value is reduced by 20%–40% compared with *ad libitum* feeding) leads to an elevation of NAD^+^ levels in most metabolically active tissues, resulting in increased SIRTs activity [11].

## 3. SIRTs in Control of Lipid and Glucose Metabolism

A growing body of literature including both *in vitro* and *in vivo* studies has implicated SIRTs in the regulation of lipid and glucose metabolism. These studies let us understand the complexity of SIRTs actions and give hope that modulation of their activity may constitute a new therapeutic strategy for the treatment of metabolic complications of obesity, such as hyperlipidemia, liver steatosis or diabetes.

### 3.1. SIRTs in Lipid Metabolism

*SIRTs* are expressed in tissues and organs involved in lipid metabolism, including liver, skeletal muscle, white (WAT) and brown (BAT) adipose tissues. In these tissues, SIRTs control lipid synthesis, storage and utilization, both directly and indirectly (via control of insulin secretion).

During fasting SIRT1 stimulates transcription of the gene encoding triglyceride lipase and subsequent lipolysis in the adipose tissue by deacetylation of peroxisome proliferator-activated receptor γ (PPARγ) co-repressors, forkhead box protein O1 (FOXO1) and PPARγ coactivator 1α (PGC1α). This process is impaired in *sirt1^−/−^* mice [12], however results of animal studies regarding *sirt1* overexpression on body weight and composition are inconsistent ([13,14] see below). It is suggested that these discrepancies may be attributed to different levels of *sirt1* expression between the transgenic animals as well as to differences between strains and species used in the experiments.

Fasting and cold exposure were found to increase expression of *SIRT2* in WAT. That resulted in the deacetylation of FOXO1 and subsequent repression of PPARγ activity, lipolysis and the release of FA. A similar effect can be obtained by administering isoproterenol, which confirms the role of adrenergic signaling in the regulation of SIRT2 expression in WAT [15]. SIRT2 may also inhibit lipogenesis by deacetylation of ACLY (ATP-citrate lyase), an enzyme crucial for FA synthesis. A deacetylated form of ACLY is then ubiquitinated and degraded while reducing lipogenesis [16].

Nutritional and thermal stress increase BAT expression of *SIRT3*, which regulates mitochondrial function and thermogenesis in brown adipocytes. In cultures of brown adipocyte precursors (HIB1B cells), overexpression of *SIRT3* resulted in increased phosphorylation of the cAMP response element-binding protein (CREB), which then directly activated PGC-1α promoter, resulting in increased expression of the gene encoding uncoupling protein 1 (UCP1) and promotion of mitochondrial respiration [17]. However, subsequent experiments showed that the protein produced on the basis of cDNA used in this experiment lacked proper deacetylase activity, so this finding should be treated with caution [18]. Moreover, *sirt3^−/−^* mice, despite mitochondrial protein hyperacetylation, showed no major disturbances of adaptive thermogenesis [19]. In contrast, livers from mice lacking *sirt3* showed higher levels of FA oxidation intermediate products and triglycerides during fasting, associated with decreased levels of FA oxidation when compared with wild-type mice. These findings are consistent with the fact that deacetylation of the long-chain acyl coenzyme A dehydrogenase by SIRT3 was found to determine proper mitochondrial FA oxidation [20].

In the liver, SIRT1 positively regulates transcription of PPARα and PGC-1α, both involved in FA catabolism. In animal models, overexpression of *sirt1* in the liver leads to the activation of PPARα targets and protects experimental animals from diet-induced hepatic steatosis [21]. Data regarding clinical consequences of *sirt1* knock-out in the liver are inconsistent, and depending on the breeding condition may result in weight gain and hepatic steatosis, or protect from fat accumulation [22,23]. Apart from regulation of PPARα-related pathways, SIRT1 may influence FA metabolism via regulation of sterol regulatory element binding protein1 (SREBP-1c) transcription factor. *SIRT1* overexpression or SIRT1 activation by Resveratrol decreases SREBP-1c acetylation *in vitro*, while *sirt1^−/−^* mice have lower SREBP-1c mRNA levels in the liver, correlating with decreased serum triglyceride concentrations [24]. Activation of SIRT1 in the liver also induces phosphorylation of AMP-activated protein kinase (AMPK), which protects against fatty acid synthase induction and lipid accumulation caused by high glucose [25].

Recently there has been a rapidly growing interest in the role of miRNAs (small endogenously expressed RNAs that regulate gene expression at a post-transcriptional level) in the regulation of lipid metabolism in different tissues. *In vitro* studies have shown that in one of the known SIRT1-targeting miRNAs, miR-34a is up-regulated in murine liver, in response to obesity. This finding suggests that when decreased by an interference with miR-34a, SIRT1 levels may be responsible for the development of obesity-associated liver steatosis [26]. SIRT1 also promotes deacetylation of liver X receptor (LXR) proteins, transcription factors that act as cholesterol sensors and regulate whole-body cholesterol and lipid homeostasis [27]. LXR deacetylation is necessary both for the activation and induction of LXR target genes, as well as their subsequent ubiquitination. Animals with *sirt1^−/−^* knock-out have reduced mRNA levels of LXR target genes, which results in impaired reverse cholesterol transport—a process by which excess cholesterol is removed from the peripheral cells and transported to the liver where it can be converted to bile and excreted [28].

In skeletal muscles, SIRT1 was found to control the switch from glucose to FA utilization. SIRT1 via deacetylation of PGC-1α stimulates β-oxidation of FA in muscle cell lines and *sirt1* knock-down results in a decrease in the expression of genes involved in this process [29,30].

There are experimental data that other SIRTs are also involved in lipid metabolism. In the fed state, SIRT4 represses FA oxidation in skeletal muscle and stimulates lipogenesis in WAT by deacetylation and repression of malonyl CoA decarboxylase, an enzyme that produces acetyl CoA from malonyl CoA. Malonyl CoA is a substrate for lipogenesis and also inhibits fat oxidation [31,32]. In turn, desuccinylation of the hydroxyacyl-coenzyme A dehydrogenase by SIRT5 represses its enzymatic activity, resulting in decreased β-oxidation of FA [33]. Finally, *in vitro* studies revealed that *sirt7* deficient animals, due to impaired management of endoplasmic reticulum stress, have increased lipogenesis in the liver. In contrast, *sirt7* up-regulation restores hepatic homeostasis in diet-induced obesity [34].

In summary, while some sirtuins promote lipolysis in WAT (SIRT1 and SIRT2), thermogenesis in BAT (SIRT3), and protect from excess lipid accumulation in skeletal muscle and the liver (SIRT1 and SIRT7), other members of SIRTs family stimulate lipogenesis and inhibit FA oxidation (SIRT4 and SIRT5). These findings may constitute the basis for studies on the use of sirtuin activating/inhibiting compounds in order to increase lipolysis and to prevent the development of hepatic steatosis in obese individuals.

### 3.2. SIRTs in Insulin Secretion and Glucose Metabolism

SIRTs are also indirectly involved in the regulation of lipid metabolism by affecting the secretion and action of insulin, which promotes FA storage in WAT and inhibits their β-oxidation in the liver and skeletal muscle.

Insulin secretion in pancreatic β cells is related to adenosine-5′-triphosphate (ATP) production. High intracellular glucose levels result in increased synthesis of ATP, which subsequently inactivates ATP-sensitive potassium channels. The reduction of potassium efflux leads to depolarization of the plasma membrane, opening the voltage-gated calcium channels and subsequent calcium influx that stimulates insulin exocytosis. The efficiency of insulin secretion can be modulated by the uncoupling protein 2 (UCP2) that regulates intracellular levels of ATP. SIRT1 decreases expression of the gene encoding UCP2 by directly binding to its promoter. Therefore, local overexpression of *sirt1* in pancreatic β cells results in enhanced insulin secretion, in response to glucose *in vitro* and improved glucose tolerance *in vivo* [35,36].

Consistently, *sirt1* knock-out by RNA interference led to decreased insulin secretion in β cell lines and its systemic depletion—to impaired glucose tolerance [37]. SIRT1, via activation of FOXO1, also participates in protecting β cells from damage-induced apoptosis. In response to toxins or to oxidative stress, FOXO1 activates expression of multiple genes to preserve insulin secretion and promote cell survival, while inhibition of SIRT1 (for example by miR-34a, whose levels increase in response to fatty acid-induced β cell dysfunction) is associated with induction of β cell apoptosis [38,39].

In contrast, SIRT4 acts as a negative regulator of insulin secretion. This mitochondrial sirtuin is selectively expressed in pancreatic β cells, were it mainly acts as a mono-ADP-ribosyltransferase. By ADP-ribosylation, SIRT4 represses the activity of glutamate dehydrogenase (GDH), an enzyme responsible for the conversion of glutamate to α-ketoglutarate, thereby promoting ATP synthesis by introducing amino acids into the tricarboxylic acid cycle. Therefore, systemic or local (for example by miR-15b) *sirt4* knock-out enhances insulin secretion in response to glucose and amino acids, while its overexpression has the opposite effect [40,41]. CR was found to decrease both SIRT1 and SIRT4 activity in the pancreas, not because of a decrease in their protein levels, but as a result of the decreased NAD/NADH ratio [42], which on the one hand reduces glucose-dependent insulin secretion and on the other hand sensitizes β cells to amino acids. This mechanism is believed to play an important role in the regulation of insulin secretion during CR that promotes deribosylation of GDH and potentates amino acid stimulated insulin secretion by β cells.

SIRTs are also involved in glycolysis and gluconeogenesis regulation. Under normal glucose availability, SIRT6 inhibits expression of multiple glycolytic genes by competing at their promoters with hypoxia induced factor 1α (HiF1α). By deacetylating histone H3 lysine 9 at promoters of Hif1α target genes, SIRT6 maintains proper glucose flux for mitochondrial respiration and prevents excessive glycolysis. In SIRT6-deficient cells, increased Hif1α activity leads to up-regulation of glycolysis and diminished mitochondrial respiration [43]. Hepatic-specific disruption of SIRT6 by miR-33 in mice results in enhanced glycolysis and triglyceride synthesis causing liver steatosis and correlates with increased triglyceride content observed in human hepatic cell lines, transfected with miR-33 [44]. SIRT1’s effect on gluconeogenesis depends on the energy resources. During short-term fasting, SIRT1 represses CRTC2 (a transcriptional coactivator for transcription factor CREB acting as a central regulator of gluconeogenic gene expression, in response to cAMP) leading to decreased gluconeogenesis. Similar effects can be obtained by interaction of SIRT1 with the signal transducer and activator of transcription 3 (STAT3) transcription factor. In contrast, when CR is prolonged, SIRT1 promotes gluconeogenesis and inhibits glycolysis by deacetylation of PGC-1α and activation of FOXO1 [45].

*SIRT1* expression may also modify insulin sensitivity; in muscle cells by transcriptional repression of the protein tyrosine phosphatase 1B gene (PTP1B, a negative regulator of the insulin signaling pathway); while in adipose tissue via an effect on glucose transporter type 4 (GLUT4) translocation, it regulates insulin-stimulated glucose uptake [45]. Moreover, by interference with the nuclear factor κB (NF-κB) signaling pathway, SIRT1 can also repress inflammatory gene expression in adipocytes and macrophages infiltrating adipose tissue, which results in an improvement of insulin signaling pathways and a reduction of hyperinsulinemia, accompanied by increased insulin sensitivity *in vivo* [46,47]. There is also evidence that SIRT1 may affect insulin signaling by deacetylation of the insulin receptor substrate (IRS-2) [48].

It can therefore be assumed that administration of sirtuins modulators (for example SIRT1 activators and/or SIRT4 inhibitors) can have a favorable effect on insulin secretion, while activation of SIRT6 may improve intracellular glucose metabolism and protect from insulin resistance.

## 4. SIRTs and Adipogenesis

Interest in SIRTs as potential targets for the treatment of obesity also results from their involvement in the regulation of adipogenesis.

PPARγ is considered a chief transcription factor responsible for promoting adipogenesis, by interacting with co-repressors of PPARγ; nuclear receptor co-repressor (N-CoR), and silencing mediator of retinoid and thyroid hormone receptors (SMRT), SIRT1 attenuates adipogenesis [12]. Consistently, overexpression of ectopic *sirt1* blocks adipogenesis in 3T3-L1 cells, a culture of mouse adipocytes was used as a model of adipocyte differentiation [49]. Additionally, via activation of the Wnt signaling pathway, SIRT1 determinates mesenchymal stem cell (MSC) differentiation towards myogenic cells, while its inhibition in MSC promotes adipogenesis [50].

One concept of obesity treatment is based on the activation of preadipocyte genes specific to BAT, which is characterized by high metabolic activity. Since SIRT1, by direct deacetylation of PPARγ, recruits the BAT program coactivator Prdm16 to PPARγ, it also plays a crucial role in the induction of genes typical for BAT and repression of visceral WAT genes associated with insulin resistance [51]. Therefore, silencing of *sirt1* in 3T3-L1 preadipocytes leads to their hyperplasia and increased expression of WAT and inflammatory markers with a parallel decrease in BAT markers [52]. Recent studies underlined the role of different miRNAs as negative regulators of SIRT1 during adipocyte differentiation, for example miR-34a, miR-146b, miR-181a, suggesting that interference with these miRNAs may constitute a therapeutic approach in the treatment of excess adiposity, as well as in the activation of brown adipocytes [26,53].

Another sirtuin family member, SIRT2, was also shown to exert an inhibitory effect on adipocyte differentiation [15]. This process is mediated by FOXO1 deacetylation and subsequent PPARγ transcriptional activity repression [54]. Therefore, *sirt2* overexpression inhibits adipogenesis, while its silencing has an opposite effect in 3T3-L1 preadipocytes. This inhibitory effect of SIRT2 on adipocyte differentiation discloses under nutritional stress suggesting that combination of SIRT2 activators with diet could provide novel therapeutic strategy for obesity. However, such compounds have not been developed yet (see below).

*In vitro* studies showed that SIRT7 is required for PPARγ expression and proper adipocyte differentiation. Unlike SIRT1 and SIRT2, its deletion or downregulation diminishes the ability of mouse embryo fibroblasts and 3T3L1 cells to undergo adipogenesis. However, its overexpression did not restore preadipocyte differentiation, suggesting that SIRT7 is required but not sufficient to perform a full program of adipogenesis. Interestingly, SIRT7 is a metabolic target for miR-93, a negative regulator of adipogenesis, whose expression is decreased in genetically obese ob/ob mice [55]. This miRNA was also found to be a key regulator of mature adipocyte turnover and its inhibition increases fat tissue formation *in vivo*.

Therefore, it can be expected that in the future activation of SIRT1 and SIRT2 or inhibition of SIRT7 by appropriate miRNA, may be a therapeutic strategy for the treatment of obesity.

## 5. Abnormal Activity of SIRTs System in Obesity

### 5.1. Animal Studies

As described above, *in vitro* studies have shown the important role of SIRT enzymes in the regulation of glucose and lipid metabolism as well as in the control of adipogenesis. *In vivo* studies, in which particular genes encoding SIRTs were either silenced or overexpressed in experimental animals, have provided further data on the role of SIRTs in the pathogenesis of obesity. Additionally, abnormalities of SIRTs’ action were also investigated in different animal models of obesity.

SIRT1 deficiency may result in different phenotypes. Complete, systemic knock-out of *sirt1* in inbred mice leads to serious developmental defects, sterility and high postnatal lethality; whereby these animals are not appropriate for the study of energy metabolism. However, some of the outbred *sirt1^−/−^* mice reached adulthood and due to the hypermetabolic state have significantly lower body weight compared to wild-type animals [56]. The heterozygous *sirt1^+/−^* mice develop and reproduce normally, and when fed a high-fat diet have increased body and liver fat content, reduced energy expenditure, elevated inflammatory parameters in sera, adipose tissue and liver that resemble the metabolic syndrome phenotype [57].

Liver-specific *sirt1* knock-down in mice on CR results in lower systemic cholesterol levels and increased liver accumulation of cholesterol and FA. However, results regarding the metabolic consequences of a high-calorie diet in these animals are contradictory. While some researchers reported that they have lower liver steatosis rates than wild-type controls bred in the same manner, others claim that liver-specific *sirt1* knock-down in mice is associated with severe liver steatosis and inflammatory infiltrations [45].

Specific genetic ablation of *sirt1* in WAT leads to obesity, increased inflammatory infiltration, and insulin resistance similar to that observed in high-fat diet induced obesity [11]. However, in a recent study, adipocyte-specific knock-out of *sirt1* in short-term observation led to an exacerbation of metabolic complications, while it improved metabolic functions long-term [58]. The authors of the study suggested that this phenomenon is related to the obesity-associated unphosporylation of PPARγ that induces a set of target genes which can promote insulin sensitivity.

In contrast, transgenic mice moderately overexpressing *sirt1* under the control of a β-actin promoter develop a phenotype similar to mice on CR. They gain less body weight and have reduced levels of serum cholesterol, glucose and insulin compared with littermate controls, and are protected against the negative metabolic consequences of a high-fat diet [13,59,60]. However, when *sirt1* is overexpressed under its own promoter, the animals, despite decreased food intake, have normal body weight due to lower energy expenditure and are not protected from diet-induced obesity [14].

Although a detailed presentation of studies on the role of SIRTs in the central regulation of appetite is beyond the scope of this paper, it should be mentioned that while animal studies suggest that peripheral actions of SIRT1 mostly promote a negative energy balance, its role in the central regulation of metabolism is less clear. On the one hand, CR leads to increased expression of *sirt1* in hypothalamic nuclei in mice, especially in the arcuate nucleus (ARC) producing anorexigenic proopiomelanocortin (POMC) and orexigenic peptides, including neuropeptide Y (NPY) and aguit-related peptide (AgRP). Consistently, targeted *sirt1* overexpression in the hypothalamus results in a similar phenotype and biochemical profile as in animals on a hypocaloric diet [23]. Overexpression of s*irt1* in mouse POMC or AgRP neurons also prevents age-associated weight gain in two different manners: in POMC neurons it stimulates energy expenditure via increased sympathetic activity in adipose tissue and in AgRP neurons, it suppresses food intake. These beneficial results are diminished in aging mice and mice consuming an high-fat, high-sucrose diet due to decreases in SIRT1 and NAD^+^ levels in the hypothalamus. This shows that age and obesity-related decline in ARC SIRT1 function contributes to a disruption of energy homeostasis [61].

On the other hand, there are studies that suggest that in the central neural system SIRT1 promotes a positive energy balance. Mice with neuron-specific *sirt1* knock-out (SINKO) are protected against the metabolic complications of a high-fat diet that include lower fasting insulin levels, improved glucose tolerance, enhanced systemic and central insulin sensitivity compared with wild-type animals, suggesting that neuronal SIRT1 negatively regulates hypothalamic insulin signaling, leading to systemic insulin resistance [62]. Consistently, acute inhibition of SIRT1 by intracerebroventricular injection of its inhibitor (Ex-527) in rodents results in long-term reduced food intake and body weight gain, which is accompanied by an increase in POMC levels and a decrease in AgRP in ARC [63]. These findings are contributed to a SIRT1-mediated reduction of FOXO-1 activity that positively regulates transcription of genes encoding AgRP and NPY and decreases expression of POMC [64]. Collectively, these results suggest that in the hypothalamus SIRT1, via its effect on FOXO1, may promote cell-specific functions that result in divergent adaptive, metabolic responses [65]. Taking the above data into account, even though approaches aimed at enhancing peripheral SIRT1 activity while reducing its central action, may seem attractive in the treatment of obesity, their use in clinical practice requires more detailed knowledge of the SIRT1 function in distinct organs and tissues.

Compared with SIRT1, knowledge about the role of other SIRTs in the pathogenesis of obesity is relatively limited. Nevertheless, recent years have brought new data from animal models suggesting that SIRT2-7 are also important in this aspect.

Systemic knock-out of *sirt2* predominantly leads to enhanced tumorogenesis in animals that is consistent with its role in cell cycle control. However, it may also play a role in obesity-associated inflammation that is believed to mediate in metabolic complication development. By deacetylation of p65 (a member of a NF-κB family), SIRT2 inhibits its translocation from the cytoplasm to nucleus, and therefore expression of NF-κB-dependent genes. Subsequently, cells from *sirt2^−/−^* animals present an excessive inflammatory response to stimuli activating the NF-κB pathway [66].

Mice lacking SIRT3 suffer from FA oxidation disorders during fasting and from impaired adaptive thermogenesis leading to intolerance of cold exposure [17]. Specific ablation of *sirt3* in hepatocytes promotes lipid accumulation, while mice that develop fatty liver disease on a high-fat diet exhibit reduced SIRT3 activity and mitochondrial protein hyperacetylation. Therefore, it is suggested that during an excess of nutrients SIRT3 may act as a guard protecting from hepatic lipotoxicity [20]. Moreover, *sirt3^−/−^* mice are unable to counteract oxidative stress, suggesting that SIRT3 may be responsible for a delay of oxidative stress-related pathologies [45].

As mentioned above, SIRT4 is a negative regulator of insulin secretion; therefore its ablation in mice results in an increased insulin burst, in response to glucose and amino acids [40]. Targeted knock-down of *sirt4* in mouse primary hepatocytes and muscle cells leads to increased expression of mitochondrial FA metabolism enzymes *in vitro*. Inhibition of SIRT4 activity *in vivo*, by administration of shRNA adenovirus, also results in increased hepatic mitochondrial and FA oxidation gene expression consistent with findings in primary hepatocytes. Taken together, these results demonstrate that SIRT4 inhibition increases the fat oxidative capacity in the liver and mitochondrial function in muscle, which might provide therapeutic benefits for diseases associated with ectopic lipid storage, such as type 2 diabetes. However, it is not clear if these effects result directly from a SIRT4 deficiency, since *sirt4* ablation is accompanied by an increase in sirt1 mRNA and protein levels, both *in vitro* and *in vivo*. This suggests that the observed changes may depend on the up-regulation of *sirt1* [67].

Germline SIRT5 deficient mice (*sirt5^−/−^*) are characterized by global protein hypersuccinylation and elevated serum ammonia during fasting; however, they do not present any overt metabolic abnormalities, under either normal or high-fat diet conditions [68]. Consistently, a dominant abnormality observed in mice overexpressing *sirt5* is up-regulated urea synthesis compared with wild-type animals [69]. These findings suggest that SIRT5, crucial for the urea cycle, is probably unnecessary for metabolic homeostasis under basal conditions.

Mice with systemic SIRT6 depletion are characterized by a loss of subcutaneous fat, lymphopenia and acute hypoglycemia leading to premature death. This phenotype resembles changes that develop during ageing and may result from impaired DNA repair and genomic instability [70]. Interestingly, mice with targeted neural *sirt6* ablation reach normal size and over time develop obesity that is accompanied by hyperacetylation of H3K9 and H3K56 (histone H3 lysine 9 and lysine 56), both implicated in the regulation of gene activity and chromatin structure in hypothalamic nuclei [71].

In turn, SIRT6 transgenic animals, despite a high-fat diet, accumulate less visceral fat, have lower low density lipoprotein cholesterol (LDL-C) and triglyceride levels as well as enhanced glucose tolerance and insulin sensitivity in the liver and skeletal muscles than wild-type controls [72,73]. These beneficial effects were associated with the down-regulation of PPARγ-responding genes, including those involved in lipid storage regulation as well as NF-κB signaling attenuation. It is therefore suggested that SIRT6 activation may be considered as a therapeutic strategy to treat metabolic diseases.

Finally, mice lacking SIRT7 are unable to maintain a constant body temperature and normal blood glucose levels under cold and starvation stress [74]. Compared with wild-type animals, *sirt7^−/−^* mice present increased expression of genes involved in gluconeogenesis and decreased expression of genes involved in lipogenesis during fasting. In turn, impaired cold resistance may be caused by a lower expression of PGC-1α, a critical regulator of adaptive thermogenesis. Moreover, *sirt7* deficient mice displayed reduced adipocyte size, stunted adipogenic gene expression, resistance to high-fat diet-induced liver steatosis and insulin resistance [75]. However, since this phenotype reminds us of the description of PPARγ^+/−^ mice, it is not clear whether abnormalities observed in *sirt7^−/−^* mice result from the lack of SIRT7 or the reduced PPARγ expression and/or activity [55].

In conclusion, animal studies have shown that, apart from SIRT5, all other SIRT are implicated in metabolic regulation and their abnormal function may contribute to the development of obesity.

### 5.2. Human Studies

The knowledge of the role of SIRTs in the regulation of metabolism comes primarily from animal studies. The number of publications on the role of SIRTs in regulating metabolism in humans is limited and can be classified into two categories: studies on *SIRTs* expression in metabolically active tissues and genetic studies concerning the association between polymorphisms in *SIRTs* and development of obesity in humans.

### 5.3. Human Obesity Associated Changes in SIRTs Expression

Human studies suggest that obesity is accompanied by alternations of SIRTs levels in distinct tissues and organs.

Transcript levels of *SIRT1* in adipose tissue and peripheral blood mononuclear cells (PBMC) are significantly lower in obese individuals, compared with normal-weight controls that can be successfully restored by weight loss obtained either by CR or bariatric surgery [8,76,77,78,79,80]. Since SIRT1 increases lipolysis and suppresses inflammatory responses, it is plausible that its decreased expression in the adipose tissue of obese individuals might be associated with excessive fat accumulation and development of obesity-related inflammation. This hypothesis is supported by the fact that the lowest *SIRT1* mRNA levels can be found in adipose tissues from obese patients diagnosed with T2DM and severe hepatic steatosis [80,81].

Weight loss is also associated with increased *SIRT3* and *SIRT6* mRNA levels in liver and subcutaneous adipose tissues of morbidly obese individuals [8]. This finding is consistent with the abovementioned animal studies, where diet-induced fatty liver disease is accompanied by reduced SIRT3 activity. While mice overexpressing *sirt6*, despite a high-fat diet, accumulate less visceral fat, have a more favorable lipid profile and enhanced glucose tolerance compared with wild-type controls [72].

*SIRT4* mRNA levels in serum and PBMC are inversely correlated with different anthropometric parameters (BMI, waist circumference, fat mass) as well as with insulin, cholesterol and triglyceride concentrations [82,83]. However, simultaneous analysis of the growth hormone/IGF-1 axis revealed that these relations are probably secondary to the low GH and IGF-1 levels observed in obese individuals, both involved in the regulation of SIRT4 status [31]. Nevertheless, according to the hypothesis that mitochondrial functions are adjusted to environmental requirements, it is suggested that low serum SIRT4 levels in obesity occur in response to calorie excess in order to decrease fat oxidative capacity in the liver and muscles, but promote excess ectopic lipid storage [82].

In contrast, mean mRNA levels of *SIRT7* are higher in adipose tissues obtained from obese individuals than in those of normal-weight controls (Kurylowicz A., unpublished) Knowledge about the role of SIRT7 in the pathogenesis of human obesity is still unclear and its expression in human adipose tissue has not been previously documented. As mentioned above, in rodents *sirt7* knock-out led to resistance to high-fat diet-induced obesity, liver steatosis and glucose intolerance; however these abnormalities may result from impaired PPARγ expression [75]. Some data also suggests an interaction between SIRT1 and SIRT7 at the molecular level, as was shown in immunoprecipitation assays and *in vivo*, where SIRT1 protein levels and enzymatic activity were increased in WAT of animals with *sirt7* knock-out [55,84]. In our work, we also observed a negative correlation between *SIRT1* and *SIRT7* mRNA levels in adipose tissues from obese individuals. Since it is suggested that SIRT7 inhibits SIRT1 auto-deacetylation and restricts its activity, the proper balance between these two sirtuins may be therefore crucial for the maintenance of metabolic homeostasis [55].

Although there is growing evidence pointing to the role of SIRTs in the regulation of different metabolic pathways in humans, the factors that control their expression in adipose tissues are less known. In our research, we detected a negative correlation between *SIRT1* and *SIRT7* mRNA levels and the levels of relevant miRNAs, the role of which in the regulation of expression of these genes had been previously demonstrated *in vitro* [26,85,86,87,88]. With respect to *SIRT1*, we observed that its lower expression in adipose tissues of obese individuals correlated negatively with levels of miR-34a (which is physiologically up-regulated in mature adipocytes), and with two miRNA that have opposite effect on adipocyte differentiation: miR-22 (inhibiting adipogenic differentiation by targeting histone deacetylase 6) and miR-181a (promoting adipocyte differentiation by inhibition of the tumor necrosis factor α pathway) [85,86,87]. In turn, in our study, *SIRT7* targeting miR-125a was under-expressed in obese individuals and this finding was consistent with animal studies where down-regulation of this miRNA in murine adipocytes was associated with insulin resistance [88]. In contrast, we found no association between CpG methylation status of the *SIRT1* and *SIRT7* regulatory regions and their expression on the mRNA level in adipose tissues, so it is possible that methylation does not play a crucial role in obesity-associated changes in *SIRT*s expression in humans (Kurylowicz A, unpublished).

### 5.4. Genetic Studies

There is also indirect evidence from genetic studies that SIRTs might participate in the development of obesity in humans, because some of their genetic variants were found to be associated with increased weight gain and metabolic complications. As in the case of *in vitro* and animal studies, the majority of genetic research concerned SIRT1 (Table 1).

A genome-wide association study performed on Scandinavian subjects with diabetes showed a strong association between body weight and a marker in the *SIRT1* locus [89]. Subsequently, several single nucleotide polymorphisms (SNPs) located in *SIRT1* had been tested for their association with obesity and metabolic syndrome components. Since *SIRT1* SNPs remain in a high linkage disequilibrium (LD), most genetic studies focused on analyzing tag SNPs that captured the information of the other linked variants.

In the initial study by Lagouge *et al*. [60], rs2273773 SNP (C/T) located in exon 5, the promoter SNP rs3740051 (A/G), and intronic variant rs2236319 (A/G) were found to be associated with energy expenditure in 123 Finish normal weight offspring of probands with T2DM. Consistently, individuals heterozygous for rs2273773 had significantly higher BMI than TT subjects in a Dutch population study of 3575 patients [90].

However, in a subsequent case-control study that analyzed two intronic tag SNPs, rs3818292 (A/G, linked to rs2273773) and rs7069102 (G/C, linked to the several *SIRT1* promoter variants and two coding SNPs in exon1), in 1068 Belgian obese subjects and 313 lean controls, the results were not so clear [91]. While the CC genotype of rs7069102 conferred lower risk of obesity, after adjustment for BMI, it was associated with increased visceral fat in men. The authors tried to explain this unexpected finding by the fact that a decrease in BMI may be caused by a loss of both lean and fat mass, as well as the observed associations representing a sex-specific effect.

Three other *SIRT1* tag SNPs (rs7895833 A/G, rs1467568 A/G and rs497849 G/A) were analyzed for their effect on body weight in a Dutch cohort of elderly subjects from the Rotterdam Study (6251 individuals) and subsequently replicated in a group of 2347 participants of the Erasmus Rucphen Family study of a wide age range [92]. In these populations, the minor alleles of rs7895833 (G) and rs1467568 (G) were associated with a decreased BMI and carriers of their combination had a lower risk of obesity (by 13%–18%) and a smaller increase in BMI over a follow-up period.

An association between the rs12413112 (G/A), rs33957861 (C/T), rs11599176 (A/G) and rs35689145 (G/A) *SIRT1* variants, all within high LD and obesity, was confirmed in 896 unrelated French cases and 532 normal-weight controls, and replicated in 154 Swedish families (732 subjects) [77]. For each of these variants, the minor allele (Table 1) was found more frequently in normal-weight individuals, suggesting a protective association. However, the intronic location of these SNPs indicates again that they are probably only tagging another causative variant. Moreover, none of these polymorphisms were found to be associated with child obesity or with *SIRT1* mRNA levels in subcutaneous adipose tissue. What is interesting is this study did not point at two other *SIRT1* SNPs: rs7069102 and rs1467586, both associated with obesity in Belgian and Dutch populations, respectively. These discrepancies can probably be explained by the lower power of the previous studies [91,92].

*SIRT1* variants have also been studied for their effect on different clinical and metabolic parameters as well as susceptibility to lifestyle modifications. In 196 German individuals from a diabetes risk population, the carriers of the A allele of intronic rs12413112 G/A SNP were found to be resistant to lifestyle-induced improvement of fasting plasma glucose, insulin sensitivity and liver steatosis [93]. No significant associations were observed between these parameters and rs730821 (A/G) SNP in *SIRT1* promoter or with the two intronic variants—rs7069102 and rs2273773 described above. Notably, none of these SNPs was associated with BMI, nor with weight in a larger cohort of German subjects, even though rs730821 SNP was in a high LD with rs7895833 A/G, which is associated with BMI in the Dutch population [92].

However, in 1279 Japanese individuals, the AA genotype of rs7895833 was a risk factor for higher body fat content and higher BMI in males as well as for higher diastolic blood pressure in females. In this study, the male GG carriers of rs7069102 had a higher body fat ratio and systolic blood pressure. For rs2273773 SNP, male individuals with TT genotype had a higher fasting glucose level and body fat ratio, while CC male carriers—higher systolic and diastolic blood pressure [94]. Interestingly, the rs7895833 (G) and rs1467568 (G) alleles, described as minors in Caucasians [92], occurred to be major variants in Japanese individuals, reflecting population differences in the distribution of *SIRT1* SNPs. Nevertheless, both studies confirmed an association of the A allele of rs7895833 and the G allele of rs7069102 with obesity.

In contrast, analysis of the same 3 SNPs in 120 obese children and 120 normal-weight controls of Turkish origin has recently revealed that the AG genotype and G allele of rs7895833 were associated with obesity, while the AA genotype and the A allele were protective [95]. Although the rs7069102 and rs2273773 variants were not associated with higher BMI in this study, the obese children with the CT genotype of rs2273773 SNP had higher insulin levels then obese carriers of the CC genotype. However, due to the low statistical strength, the results of this study should be treated with caution.

Finally, in a prospective study where 1390 Dutch individuals had been in an 18-year follow-up, the C allele of the *SIRT1* rs12778366 (C/T) SNP was associated with a significantly reduced mortality in obese/overweight subjects and better glucose tolerance in 535 male individuals [96]. Two other SNPs analyzed in this study (rs7069102 and rs2273773) had no effect on mortality or glucose tolerance.

Unfortunately, few of the above described association studies have been complemented by functional data, which would explain the relationship of the *SIRT1* SNPs with obesity. In this respect, the work of Zarrabeitia *et al.* [97] is exceptional. The authors not only found that the T allele of the rs12049646 variant is associated with increased BMI in Hispanic Caucasians, but also that the protecting from obesity C allele of this SNP shows stronger binding properties of nuclear proteins. 

Apart from the case-control studies testing the effect of *SIRT1* polymorphisms on genetic predisposition to obesity, there is also evidence for an association of different *SIRTs* SNPs with prevalence of obesity-related complications.

In a study performed on 1770 healthy unrelated Caucasians from Austria, rs12413112 *SIRT1* SNP was associated with a mean common carotid intima-media thickness (cIMT), while 5 other SNPs (namely rs3740051 (A/G), rs2236319 (A/G), rs10823108 (G/A) as well as the abovementioned rs2273773 and rs1467568) showed a differential effect on IMT in men and women: with decreasing IMT for each minor allele for men and increasing IMT for a minor allele in women [98]. The sex-dependent effect of *SIRT* polymorphisms on atherosclerosis was also observed in a study of 1356 stroke-free North Americans, where male carriers of the *SIRT3* rs12363280 G and rs4980329 T alleles had higher grey scale median (GSM), an indicator of plaque morphology and predictor of stroke, while women homozygous for *SIRT3* rs3825075 T allele had lower cIMT [99,100]. The authors of this study explained the different effects of carotid atherosclerosis in females and males by a distinct influence of sex hormones in the regulation of atherosclerosis-related genes.

In the same cohort, rs471203 (A/G) and rs12216101 (G/T) SNPs in *SIRT5* and the T allele of the *SIRT6* rs107251(C/T) SNP were associated with an increased number of carotid plaques as well as with the total carotid plaque area. Moreover, carriers of the *SIRT3* rs12363280(C/G) C variant ha lower cIMT, while the smoking carriers of the *SIRT2* rs4802998 (A/G) G allele had elevated cIMT [99,100,101].

Regarding coronary artery disease, several *SIRT1* promoter polymorphisms potentially changing the putative transcription binding sites were exclusively present in patients with myocardial infarction, not in the healthy controls without atherosclerosis [102]. Subsequently, the G variant of the rs7069102 SNP and T allele of rs2273773 were significantly more frequent in patients with CVD compared with a control group in a Turkish population, and the rs7069102 T allele carriers had higher plasma SIRT1 concentration [103].

The *SIRT1* tagging SNPs (rs7895833 A/G, rs1467568 A/G, and rs497849 G/A) were also assessed in the Rotterdam Study cohort (4573 individuals, among them 413 were diagnosed with T2DM and 378 developed T2DM during the follow-up) for their association with type 2 diabetes. Although none of them were directly associated with a risk of T2DM, diabetic carriers of the haplotype rs7895833A rs146568G rs497849G had 1.5 times higher mortality risk compared with non-carriers, which further increased among smokers and individuals with low niacin intake [92]. Other *SIRT1* variants, G allele of rs7896005 (A/G) SNP (tagged to promoter rs3758391 SNP that alters the putative p53 binding site) and T allele of rs10509291 (A/T), were found to be associated with a risk of T2DM and a decrease in acute insulin secretion in 3501 Pima Indians; however, this association was not confirmed in another group of 3003 Native Americans. Notably, none of the tag SNPs analyzed in this study were associated with BMI or body composition [79]. Moreover, the rs3758391 C variant, which is in LD with rs7896005 G allele, was found to protect from T2DM in Mexicans [104]. In three independent Japanese populations with T2DM (1502 cases and 1740 controls), the *SIRT1* rs2236319 (A/G), rs10823108 (A/G), rs3818292 (A/G) and rs4746720 (C/T) variants were associated with a risk of diabetes [105], while selected SNPs in *SIRT2*, *SIRT3*, *SIRT4*, *SIRT5* and *SIRT6* were not.

The fact that there are only a few reports about a relationship between *SIRT1* polymorphisms and the development of hyperlipidemia makes it difficult to draw any decisive conclusions. The C allele of rs2273773 SNP was found to be associated with higher serum levels of total and LDL cholesterol in Japanese male hemodialysis patients [106]. In contrast, the TT genotype of rs2273773 and the GG genotype of rs7895833 were associated with higher diastolic blood pressure, higher total cholesterol (TC) and LDL-C levels in a small study conducted in 70 Egyptian patients. The authors of this study suggested that the carriers of those genotypes might have reduced activity of SIRT1 and therefore decreased activity of LXR (protecting the organism from cholesterol overload, reducing cholesterol loading in macrophages, and protecting from atherosclerosis) [107].

In summary, despite extensive research, it is difficult to formulate firm conclusions regarding associations of distinct SNPs in genes encoding SIRTs with obesity and related complications. The first reason is the low replication rate among the studies performed in different populations. Secondly, lack of knowledge about the functional consequences of the studied polymorphisms makes the results difficult to interpret.

## 6. Sirtuins as Targets for Obesity Treatment

Given their role in the regulation of lipid and glucose metabolism, adipogenesis and appetite control SIRTs constitute promising targets for novel therapies of obesity and associated metabolic disorders. However, the discovery of a single compound that would be able to activate some SIRTs isoforms and inhibit others is still a challenge. Another difficulty is to obtain tissue action specificity for these compounds, since SIRTs activity may depend on cell type and environmental factors.

Possible applications of SIRTs’ inhibitors include, among others, treatment of cancer, immunodeficiency virus infection or neurodegenerative disorders. Until now, the only aspects in which SIRTs inhibitors could be used to treat obesity-associated metabolic disorders was inducing favorable changes in body composition. SIRT1 inhibiting compounds, such as splitomycin, suramin, salermide, EX-527 or sirtinol, can be used to increase the amount of skeletal muscle. This concept is based on animal studies where *sirt1^−/−^* mice display higher muscle growth compared with wild-type animals and mice with muscle specific *sirt1* overexpression [108]. However, SIRT1 inhibitors have not been tested for that purpose in humans.

The first invented compound that was able to activate SIRT1 in a way that mimics CR was the polyphenol Resveratrol (RSV) [109]. *In vitro* studies demonstrated that RSV is able to successfully inhibit preadipocyte maturation and induce adipocyte apoptosis [110]. However, the pro-apoptotic proprieties of RSV were observed predominantly in concentrations that may be difficult to obtain with its systemic administration *in vivo*. Therefore, this effect of RSV on adipose tissue can be difficult to apply in clinical practice [111]. The potential anti-obesity effects of RSV may also result from its influence on lipid metabolism in the liver and skeletal muscle. In skeletal muscle, by activation of PGC-1α, RSV stimulates mitochondrial activity, including β-oxidation of FA. Its administration to rodents on high fat diet protects from intramuscular lipid accumulation and insulin resistance [60,112]. Similarly in the liver, by activation of the AMPK-SIRT1 axis leading to increased β-oxidation of FA and to decreased lipogenesis, RSV prevents rodents from steatosis induced by a high fat or high calorie diet [113,114,115]. A composition containing RSV, leucine, β-hydroxymethylbutrate (HMB) and keto-isocaproic acid, has been recently patented to synergistically activate SIRT1 and SIRT3 in order to induce FA oxidation and mitochondrial biogenesis. This combination when tested on 3LT3-L1 preadipocytes was more effective in the activation of SIRT1 than RSV alone, but was also able to activate SIRT3. In c57/BL6 mice, treatment with a combination of low doses of RSV with either HMB or leucine resulted in reduction of body weight and improvement of body composition accompanied by increased insulin sensitivity. In turn, a combination of RSV with HMB and metformin was highly effective in increasing myotube FA oxidation [108]. Summarizing, in rodents RSV has proved to be effective in the reduction of adipose tissue content by inhibiting the fat accumulation processes and stimulating the lipolytic pathways [60,112]. Nevertheless, it is still being investigated whether the results of *in vivo* studies can be extrapolated to humans.

A reformulated version of RSV (resVida^®^), with improved bioavailability, when administered to 11 obese men for 30 days exerted several favorable metabolic effects similar to those that can be obtained by CR or increased physical activity. These changes included a reduction of blood pressure, hepatic lipid content as well as serum glucose, triglyceride and inflammatory marker levels with a parallel improvement in skeletal muscle mitochondrial function [116]. RSV treatment also changed the subcutaneous adipose tissue morphology and function. It increased the number of small adipocytes and caused up-regulation of genes involved in lipid breakdown by autophagy [117]. What is important, the compound was well tolerated at the tested concentration and no adverse events were reported.

Another micronized formulation of RSV, SRT501, via activation of the similar set of genes as in the case of CR, was able to counteract the negative consequences of a high calorie diet in mice as well as lower glucose levels and increase insulin sensitivity in patients with T2DM [118,119]. This effect of RSV was confirmed by a recent study where its 12 week-long administration to 10 subjects with T2DM resulted in an increase of SIRT1 and AMPK expression in muscles accompanied by an increase of the basic metabolic rate [120]. The same treatment in patients with non-alcoholic fatty liver disease reduced the alanine transaminase level and hepatic steatosis but had no beneficial effect on anthropometric parameters, markers of insulin resistance, lipid profile or blood pressure [121].

However, there are also clinical trials that question the effectiveness of RSV in obesity treatment. In a randomized, placebo-controlled, double-blinded study on 24 obese but otherwise healthy men, 4-week-long administration of RSV had no significant effect on insulin sensitivity, blood pressure, basic metabolic rate, body composition or inflammatory parameters [122]. While administration of RSV in the treatment of obesity can be considered an encouraging therapeutic approach, its prophylactic administration to individuals with normal BMI does not seem to be founded. Twelve-week-long RSV supplementation to non-obese, postmenopausal women with normal glucose tolerance did not affect body composition, basic metabolic rate or plasma levels of metabolic and inflammatory markers [123]. Major limitations of the abovementioned studies are the small numbers of participants and relatively short follow-up time; therefore, further studies to investigate the long-term and dose-dependent metabolic effects of RSV supplementation on larger cohorts are needed.

Compounds other than resveratrol also proved their effectiveness in activating SIRT1. One example is SRT2104, a compound with anti-inflammatory properties that improves glucose homeostasis and increases insulin sensitivity in animal models; however, its administration to patients with T2DM did not lead to any consistent, dose-related changes in glucose or insulin levels [124]. Another example of small molecule activators of SIRT1 is SRT1720, which is able to increase deacetylation of SIRT1 substrates *in vitro* and was successfully applied *in vivo* to treat insulin resistance in several animal models of type 2 diabetes: diet induced obesity (DIO) mice, genetically obese ob/ob mice, and Zucker fa/fa rats in a concentration 10-fold lower than SRT501 [59,118,125]. Apart from a favorable effect on glucose metabolism, by decreasing lipogenic gene expression, SRT1720 was effective in the treatment of animal models of liver steatosis [126]. However, there are also studies that question the beneficial effect of SRT1720 on metabolic parameters in animals fed a high-fat diet [127]. Moreover, in biochemical assays with native substrates and in biophysical studies, RSV and other SIRT1 activators (SRT1720, SRT2183, SRT1460) were found not to activate SIRT1 directly. It is postulated that indirect activation of RSV is mediated by the activation of AMPK, which increases intracellular NAD^+^ levels and thus induces deacetylation of SIRT1 targets [128]. However, there is also data demonstrating the direct interaction of RSV derivates with the SIRT1 enzyme molecule, since SIRT1 mutations can significantly reduce their activity [116]. Apart from the controversy regarding the mechanism of their action, another issue that may raise concerns with the use of RSV derivates in everyday practice is their target promiscuity that may result in unexpected adverse effects [129]. As a result, SIRTs modulators are still under consideration before they can be approved for routine treatment of obesity and metabolic disorders.

Recently, there has been rapidly growing interest in the role of miRNAs in fat cell development and obesity and there is also evidence that miRNA play a role in the regulation of SIRTs activity [26,85,86,87,88]. As mentioned above, SIRT-targeting miRNAs were found to be crucial for the regulation of adipogenesis and determination of MSCs differentiation towards preadipocytes (e.g., miR-34a, miR-22, miR-93, miR-146b, miR-181a) as well as lipid metabolism (miR-33, miR-34a), insulin secretion (miR-15b) and sensitivity (miR-125a), and their expression profile differs between tissues obtained from obese and normal-weight individuals [26,41,44,53,85,86,87,88]. Therefore, one can assume that strategies based on modifying the action of SIRTs by specific miRNAs may also be effective in treating obesity. However, these studies are still at a preliminary (*in vitro*) stage and it is hard to predict how interference with these miRNAs (e.g., by antisense oligonucleotide constructs—antagomiRs) may affect human metabolism *in vivo*. Another challenge is to develop safe and efficient methods of delivering miRNA-targeting therapeutics to target tissues and avoid side effects resulting from their systemic action. Finally, at this moment it is difficult to predict how many different cellular pathways may be disturbed by the action of a single miRNA, since each miRNA may target multiple mRNAs and the types of these interactions also depend on the cellular context in which they occurs.

## 7. Final Remarks and Conclusions

The more is known about the effects of SIRTs on energy balance, lipid and glucose metabolism, adipogenesis regulation as well as their impaired activity in animal and human obesity, the more attractive is the idea that their activators and inhibitors may be useful in the treatment of obesity and associated complications. Data from transgenic animal studies that proved that there are benefits derived from the activation of particular SIRT enzymes is especially encouraging. However, one should remember that SIRTs’ activities are not limited to the metabolism regulation and include, among others, control of longevity, oncogenesis as well as neurological and cardiovascular functions. Moreover, since SIRTs’ action can be tissue specific, only targeted therapeutic approaches may be efficient and enable avoiding potentially deleterious and counterproductive side-effects of global SIRTs activation/inactivation. Therefore, we still cannot commonly use compounds regulating SIRTs’ action in the treatment of human obesity. However, many of these compounds are under clinical evaluation and it is very likely that in the near future new therapeutic strategies aimed at selective and tissue-specific modulation of SIRTs’ activity will be registered for the treatment of obesity and its complications.

## Figures and Tables

**Table 1:** Associations of single nucleotide polymorphisms in genes encoding sirtuins (*SIRTs*) with obesity and related complications.

Gene	Polymorphism	Allele/Genotype	Association	Population	References
*SIRT1*	rs2273773 (T/C)	C	Lower intima-media thickness in men	1770 Austrian Caucasians	[98]
		C	Higher intima-media thickness in women	1770 Austrian Caucasians	[98]
		C	Higher TC and LDL-C levels in male hemodialysis patients	219 Japanese hemodialysis patients, 803 control subjects	[106]
		T	Associated with cardiovascular diseases	278 Turkish patients with CVD 135 controls	[103]
		CT	Higher BMI compared to TT homozygotes	3575 Dutch Caucasians	[90]
		CT	Higher insulin levels	120 obese Turkish children 120 lean controls	[95]
		CC	Higher systolic and diastolic blood pressure in men	1279 Japanese	[94]
		TT	Higher fat content and higher fasting glucose in men	1279 Japanese	[94]
		TT	Higher diastolic blood pressure and higher TC and LDL-C levels	70 Egyptian	[107]
		No association with obesity and susceptibility to lifestyle modification		196 German Caucasians	[93]
		No influence on mortality and on glucose tolerance in obese individuals		1390 Dutch Caucasians	[96]
	rs7069102 (G/C)	G	Associated with CVD	278 Turkish patients with CVD 135 controls	[103]
		CC	Lower risk of obesity but higher visceral fat Content in men	1068 obese subjects, 313 lean controls (Belgian Caucasians)	[91]
		GG	Higher fat content and higher systolic blood pressure in men	1279 Japanese	[94]
		No association with obesity		896 obese subjects, 532 lean controls (French Caucasians) 154 Swedish families (732 subjects) 120 obese Turkish children and 120 lean controls	[77,95]
		No association with obesity and susceptibility to lifestyle modification		196 German Caucasians	[93]
		No influence on mortality and on glucose tolerance in obese individuals		1390 Dutch Caucasians	[96]
	rs7895833 (A/G)	G	Lower BMI	8598 Dutch Caucasians	[92]
		G	Higher BMI	120 obese Turkish children 120 lean controls	[95]
	rs7895833 (A/G)	A	Increased mortality in diabetic patients (in a haplotype with rs1467568G/ rs497849G)	8598 Dutch Caucasians	[92]
		AA	higher BMI and higher fat content in men	1279 Japanese	[94]
		AA	higher diastolic blood pressure in women	1279 Japanese	[94]
		AG	Higher BMI	120 obese Turkish children 120 lean controls	[95]
		GG	Higher diastolic blood pressure and higher TC and LDL-C levels	70 Egyptians	[107]
		No association with BMI and fat content		3501 Pima Indians 3003 Native Americans	[79]
	rs1467568 (A/G)	G	lower BMI	8598 Dutch Caucasians	[92]
		G	Increased mortality in diabetic patients (in a haplotype with rs7895833A/rs497849G)	8598 Dutch Caucasians	[92]
		G	Lower intima-media thickness in men	1770 Austrian Caucasians	[98]
		G	Higher intima-media thickness in women	1770 Austrian Caucasians	[98]
		No association with obesity		896 obese subjects, 532 lean controls (French Caucasians) 154 Swedish families (732 subjects)	[77]
	rs12413112 (G/A)	A	Higher BMI	896 obese subjects, 532 lean controls (French Caucasians) 154 Swedish families (732 subjects)	[77]
		A	Lower energy expenditure and resistance to lifestyle interventions	196 German Caucasians	[93]
		A	Higher mean common intima-media thickness	1770 Austrian Caucasians	[98]
		No association with BMI and weight		1279 Japanese	[94]
	rs33957861 (C/T)	T	Higher BMI	896 obese subjects, 532 lean controls (French Caucasians) 154 Swedish families (732 subjects)	[77]
	rs11599176 (A/G)	G	Higher BMI	896 obese subjects, 532 lean controls (French Caucasians) 154 Swedish families (732 subjects)	[77]
	rs35689145 (G/A)	A	Higher BMI	896 obese subjects, 532 lean controls (French Caucasians) 154 Swedish families (732 subjects)	[77]
	rs730821 (A/G)	No association with BMI and weight		1279 Japanese	[94]
	rs12778366 (C/T)	C	Reduced mortality in obese/overweight individuals	1390 Dutch Caucasians	[96]
		C	Better glucose tolerance in men	1390 Dutch Caucasians	[96]
	rs12049646 (C/T)	T	Higher BMI in men	1802 Spanish Caucasians	[97]
	rs3740051 (A/G)	G	Lower intima-media thickness in men	1770 Austrian Caucasians	[98]
		G	Higher intima-media thickness in women	1770 Austrian Caucasians	[98]
	rs2236319 (A/G)	G	Lower intima-media thickness in men	1770 Austrian Caucasians	[98]
		G	Higher intima-media thickness in women	1770 Austrian Caucasians	[98]
		A	Associated with diabetic nephropathy	1502 Japanese patients with T2DM 1740 controls	[105]
	rs10823108 (G/A)	A	Lower intima-media thickness in men	1770 Austrian Caucasians	[98]
		A	Higher intima-media thickness in women	1770 Austrian Caucasians	[98]
		G	Associated with diabetic nephropathy	1502 Japanese patients with T2DM 1740 controls	[105]
	rs4746720 (T/C)	T	Associated with diabetic nephropathy	1502 Japanese patients with T2DM 1740 controls	[105]
		No association with BMI and fat content		3501 Pima Indians 3003 Native Americans	[79]
	rs497849 (G/A)	G	Increased mortality in diabetic patients (in a haplotype with rs1467568G/rs7895833A)	8598 Dutch Caucasians	[92]
	rs10509291 (T/A)	T	Associated with type 2 diabetes	3501 Pima Indians	[79]
		T	Not associated with type 2 diabetes	3003 Native Americans	[79]
		No association with BMI and fat content		6504 North Americans	[79]
	rs7896005 (G/A)	G	Associated with type 2 diabetes	3501 Pima Indians	[79]
		G	Not associated with type 2 diabetes	3003 Native Americans	[79]
	–	No association with BMI and fat content		6504 North Americans	[79]
	rs3758391 (C/T)	C	Protects from type 2 diabetes	519 Mexican patients with T2DM 389 Mexican patients with MS 547 Mexican controls	[104]
	rs3818292 (A/G)	A	Associated with diabetic nephropathy	1502 Japanese patients with T2DM 1740 controls	[105]
*SIRT2*	rs4802998 (A/G)	G	Higher intima-media thickness	1356 North Americans	[100]
*SIRT3*	rs12363280 (C/G)	G	Higher grey scale median indicator of plaque morphology and a predictor of stroke	1356 North Americans	[99]
		C	Lower intima media thickness	1356 North Americans	[100]
	rs4980329 (T/C)	T	Higher grey scale median—an indicator of plaque morphology and a predictor of stroke	1356 North Americans	[99]
	rs3825075 (T/C)	TT	Lower intima media thickness in women	1356 North Americans	[100]
*SIRT5*	rs4712032 (A/G)	G	Increased number of carotid plaques	1356 North Americans	[99]
	rs12216101 (G /T)	G	Increased number of carotid plaques	1356 North Americans	[99]
*SIRT6*	rs107251 (C/T)	T	Increased number of carotid plaques	1356 North Americans	[99,101]
	rs3760905 (G/T)	T	Increased number of carotid plaques	1356 North Americans	[99,101]

BMI—Body mass index; CVD—cardiovascular diseases; LDL-C—low density lipoproteins cholesterol; MS—Metabolic syndrome; T2DM—Type 2 diabetes mellitus; TC—Total cholesterol.

## References

[1] Klaus J.R., Hurwitz B.E., Llabre M.M., Skyler J.S., Goldberg R.B., Marks J.B., Bilsker M.S., Schneiderman N. (2009). Central obesity and insulin resistance in the cardiometabolic syndrome: Pathways to preclinical cardiovascular structure and function. J. Cardiometab. Syndr..

[2] Sanders B.D., Jackson B., Marmorstein R. (2010). Structural basis for sirtuin function: What we know and what we don’t. Biochim. Biophys. Acta.

[3] Haigis M.C., Guarente L.P. (2006). Mammalian sirtuins—Emerging roles in physiology, aging, and calorie restriction. Genes Dev..

[4] Haigis M.C., Sinclair D.A. (2010). Mammalian sirtuins: Biological insights and disease relevance. Annu. Rev. Pathol..

[5] Wang F., Nguyen M., Qin F.X., Tong Q. (2007). SIRT2 deacetylates FOXO3a in response to oxidative stress and caloric restriction. Aging Cell.

[6] Parihar P., Solanki I., Mansuri M.L., Parihar M.S. (2015). Mitochondrial sirtuins: Emerging roles in metabolic regulations, energy homeostasis and diseases. Exp. Gerontol..

[7] Zakhary S.M., Ayubcha D., Dileo J.N., Jose R., Leheste J.R., Horowitz J.M., Torres G. (2010). Distribution analysis of deacetylase SIRT1 in rodent and human nervous systems. Anat. Rec..

[8] Moschen A.R., Wieser V., Gerner R.R., Bichler A., Enrich B., Moser P., Ebenbichler C.F., Kaser S., Tilg H. (2013). Adipose tissue and liver expression of SIRT1, 3, and 6 increase after extensive weight loss in morbid obesity. J. Hepatol..

[9] Caton P.W., Richardson S.J., Kieswich J., Bugliani M., Holland M.L., Marchetti P., Morgan N.G., Yaqoob M.M., Holness M.J., Sugden M.C. (2013). Sirtuin 3 regulates mouse pancreatic beta cell function and is suppressed in pancreatic islets isolated from human type 2 diabetic patients. Diabetologia.

[10] Acs Z., Bori Z., Takeda M., Osvath P., Berkes I., Taylor A.W., Yang H., Radak Z. (2014). High altitude exposure alters gene expression levels of DNA repair enzymes, and modulates fatty acid metabolism by SIRT4 induction in human skeletal muscle. Respir. Physiol. Neurobiol..

[11] Chalkiadaki A., Guarente L. (2012). Sirtuins mediate mammalian metabolic responses to nutrient availability. Nat. Rev. Endocrinol..

[12] Picard F., Kurtev M., Chung N., Topark-Ngarm A., Senawong T., Machado De Oliveira R., Leid M., McBurney M.W., Guarente L. (2004). SIRT1 promotes fat mobilization in white adipocytes by repressing PPAR-γ. Nature.

[13] Bordone L., Cohen D., Robinson A., Motta M.C., van Veen E., Czopik A., Steele A.D., Crowe H., Marmor S., Luo J. (2007). SIRT1 transgenic mice show phenotypes resembling calorie restriction. Aging Cell.

[14] Banks A.S., Kon N., Knight C., Matsumoto M., Gutiérrez-Juárez R., Rossetti L., Gu W., Accili D. (2008). SirT1 gain of function increases energy efficiency and prevents diabetes in mice. Cell Metab..

[15] Wang F., Tong Q. (2009). SIRT2 suppresses adipocyte differentiation by deacetylating FOXO1 and enhancing FOXO1’s repressive interaction with PPARgamma. Mol. Biol. Cell.

[16] Lin R., Tao R., Gao X., Li T., Zhou X., Guan K.L., Xiong Y., Lei Q.Y. (2013). Acetylation stabilizes ATP-citrate lyase to promote lipid biosynthesis and tumor growth. Mol. Cell.

[17] Shi T., Wang F., Stieren E., Tong Q. (2005). SIRT3, a mitochondrial sirtuin deacetylase, regulates mitochondrial function and thermogenesis in brown adipocytes. J. Biol. Chem..

[18] Jin L., Galonek H., Israelian K., Choy W., Morrison M., Xia Y., Wang X., Xu Y., Yang Y., Smith J.J. (2009). Biochemical characterization, localization, and tissue distribution of the longer form of mouse SIRT3. Protein Sci..

[19] Lombard D.B., Alt F.W., Cheng H.L., Bunkenborg J., Streeper R.S., Mostoslavsky R., Kim J., Yancopoulos G., Valenzuela D., Murphy A. (2007). Mammalian Sir2 homolog SIRT3 regulates global mitochondrial lysine acetylation. Mol. Cell. Biol..

[20] Hirschey M.D., Shimazu T., Goetzman E., Jing E., Schwer B., Lombard D.B., Grueter C.A., Harris C., Biddinger S., Ilkayeva O.R. (2010). SIRT3 regulates mitochondrial fatty-acid oxidation by reversible enzyme deacetylation. Nature.

[21] Pfluger P.T., Herranz D., Velasco-Miguel S., Serrano M., Tschöp M.H. (2008). SIRT1 protects against high-fat diet-induced metabolic damage. Proc. Natl. Acad. Sci. USA.

[22] Purushotham A., Schug T.T., Xu Q., Surapureddi S., Guo X., Li X. (2009). Hepatocyte-specific deletion of SIRT1 alters fatty acid metabolism and results in hepatic steatosis and inflammation. Cell Metab..

[23] Chen D., Bruno J., Easlon E., Lin S.J., Cheng H.L., Alt F.W., Guarente L. (2008). Tissue-specific regulation of SIRT1 by calorie restriction. Genes Dev..

[24] Wang G.L., Fu Y.C., Xu W.C., Feng Y.Q., Fang S.R., Zhou X.H. (2009). Resveratrol inhibits the expression of SREBP1 in cell model of steatosis via SIRT1-FOXO1 signaling pathway. Biochem. Biophys. Res. Commun..

[25] Hou X., Xu S., Maitland-Toolan K.A., Sato K., Jiang B., Ido Y., Lan F., Walsh K., Wierzbicki M., Verbeuren T.J. (2008). SIRT1 regulates hepatocyte lipid metabolism through activating AMP-activated protein kinase. J. Biol. Chem..

[26] Alexander R., Lodish H., Sun L. (2011). MicroRNAs in adipogenesis and as therapeutic targets for obesity. Expert Opin. Ther. Targets.

[27] Li X., Zhang S., Blander G., Tse J.G., Krieger M., Guarente L. (2007). SIRT1 deacetylates and positively regulates the nuclear receptor LXR. Mol. Cell.

[28] Groen A.K., Oude Elferink R.P., Verkade H.J., Kuipers F. (2004). The ins and outs of reverse cholesterol transport. Ann. Med..

[29] Gerhart-Hines Z., Rodgers J.T., Bare O., Lerin C., Kim S.H., Mostoslavsky R., Alt F.W., Wu Z., Puigserver P. (2007). Metabolic control of muscle mitochondrial function and fatty acid oxidation through SIRT1/PGC-1α. EMBO J..

[30] Lomb D.J., Laurent G., Haigis M.C. (2010). Sirtuins regulate key aspects of lipid metabolism. Biochim. Biophys. Acta.

[31] Savastano S., di Somma C., Colao A., Barrea L., Orio F., Finelli C., Pasanisi F., Contaldo F., Tarantino G. (2015). Preliminary data on the relationship between circulating levels of Sirtuin 4, anthropometric and metabolic parameters in obese subjects according to growth hormone/insulin-like growth factor-1 status. Growth Horm. IGF Res..

[32] Laurent G., German N.J., Saha A.K., de Boer V.C., Davies M., Koves T.R., Dephoure N., Fischer F., Boanca G., Vaitheesvaran B. (2013). SIRT4 coordinates the balance between lipid synthesis and catabolism by repressing malonyl CoA decarboxylase. Mol. Cell.

[33] Park J., Chen Y., Tishkoff D.X., Peng C., Tan M., Dai L., Xie Z., Zhang Y., Zwaans B.M., Skinner M.E. (2013). SIRT5-mediated lysine desuccinylation impacts diverse metabolic pathways. Mol. Cell.

[34] Cioffi M., Vallespinos-Serrano M., Trabulo S.M., Fernandez-Marcos P.J., Firment A.N., Vazquez B.N., Vieira C.R., Mulero F., Camara J.A., Cronin U.P. (2015). MiR-93 Controls Adiposity via Inhibition of Sirt7 and Tbx3. Cell Rep..

[35] Bordone L., Motta M.C., Picard F., Robinson A., Jhala U.S., Apfeld J., McDonagh T., Lemieux M., McBurney M., Szilvasi A. (2006). SIRT1 regulates insulin secretion by repressing UCP2 in pancreatic β cells. PLoS Biol..

[36] Bordone L., Motta M.C., Picard F., Robinson A., Jhala U.S., Apfeld J., McDonagh T., Lemieux M., McBurney M., Szilvasi A. (2015). Correction: SIRT1 regulates insulin secretion by repressing UCP2 in pancreatic β cells. PLoS Biol..

[37] Moynihan K.A., Grimm A.A., Plueger M.M., Bernal-Mizrachi E., Ford E., Cras-Méneur C., Permutt M.A., Imai S. (2005). Increased dosage of mammalian Sir2 in pancreatic beta cells enhances glucose-stimulated insulin secretion in mice. Cell Metab..

[38] Kitamura Y.I., Kitamura T., Kruse J.P., Raum J.C., Stein R., Gu W., Accili D. (2005). FOXO1 protects against pancreatic beta cell failure through NeuroD and MafA induction. Cell Metab..

[39] Hughes K.J., Meares G.P., Hansen P.A., Corbett J.A. (2011). FoxO1 and SIRT1 regulate β-cell responses to nitric oxide. J. Biol. Chem..

[40] Ahuja N., Schwer B., Carobbio S., Waltregny D., North B.J., Castronovo V., Maechler P., Verdin E. (2007). Regulation of insulin secretion by SIRT4, a mitochondrial ADP-ribosyltransferase. J. Biol. Chem..

[41] Lang A., Grether-Beck S., Singh M., Kuck F., Jakob S., Kefalas A., Altinoluk-Hambüchen S., Graffmann N., Schneider M., Lindecke A. (2016). MicroRNA-15b regulates mitochondrial ROS production and the senescence-associated secretory phenotype through sirtuin 4/SIRT4. Aging.

[42] Bordone L., Guarente L. (2007). Sirtuins and β-cell function. Diabetes Obes. Metab..

[43] Zhong L., D’Urso A., Toiber D., Sebastian C., Henry R.E., Vadysirisack D.D., Guimaraes A., Marinelli B., Wikstrom J.D., Nir T. (2010). The histone deacetylase SIRT6 regulates glucose homeostasis via Hif1α. Cell.

[44] Fernández-Hernando C., Suárez Y., Rayner K.J., Moore K.J. (2011). MicroRNAs in lipid metabolism. Curr. Opin. Lipidol..

[45] Nogueiras R., Habegger K.M., Chaudhary N., Finan B., Banks A.S., Dietrich M.O., Horvath T.L., Sinclair D.A., Pfluger P.T., Tschöp M.H. (2012). Sirtuin 1 and sirtuin 3: Physiological modulators of metabolism. Physiol. Rev..

[46] Yoshizaki T., Milne J.C., Imamura T., Schenk S., Sonoda N., Babendure J.L., Lu J.C., Smith J.J., Jirousek M.R., Olefsky J.M. (2009). SIRT1 exerts anti-inflammatory effects and improves insulin sensitivity in adipocytes. Mol. Cell. Biol..

[47] Yoshizaki T., Schenk S., Imamura T., Babendure J.L., Sonoda N., Bae E.J., Oh D.Y., Lu M., Milne J.C., Westphal C. (2010). SIRT1 inhibits inflammatory pathways in macrophages and modulates insulin sensitivity. Am. J. Physiol. Endocrinol. Metab..

[48] Zhang J. (2007). The direct involvement of Sirt1 in insulin-induced insulin receptor substrate-2 tyrosine phosphorylation. J. Biol. Chem..

[49] Puri N., Sodhi K., Haarstad M., Kim D.H., Bohinc S., Foglio E., Favero G., Abraham N.G. (2012). Heme induced oxidative stress attenuates sirtuin1 and enhances adipogenesis in mesenchymal stem cells and mouse pre-adipocytes. J. Cell. Biochem..

[50] Zhou Y., Zhou Z., Zhang W., Hu X., Wei H., Peng J., Jiang S. (2015). SIRT1 inhibits adipogenesis and promotes myogenic differentiation in C3H10T1/2 pluripotent cells by regulating Wnt signaling. Cell Biosci..

[51] Qiang L., Wang L., Kon N., Zhao W., Lee S., Zhang Y., Rosenbaum M., Zhao Y., Gu W., Farmer S.R. (2012). Brown remodeling of white adipose tissue by SIRT1-dependent deacetylation of PPAR-γ. Cell.

[52] Abdesselem H., Madani A., Hani A., Al-Noubi M., Goswami N., Ben Hamidane H., Billing A.M., Pasquier J., Bonkowski M.S., Halabi N. (2016). SIRT1 limits adipocyte hyperplasia through c-Myc inhibition. J. Biol. Chem..

[53] Ahn J., Lee H., Jung C.H., Jeon T.I., Ha T.Y. (2013). MicroRNA-146b promotes adipogenesis by suppressing the SIRT1-FOXO1 cascade. EMBO Mol. Med..

[54] Jing E., Gesta S., Kahn C.R. (2007). SIRT2 regulates adipocyte differentiation through FOXO1 acetylation/deacetylation. Cell Metab..

[55] Shin J., He M., Liu Y., Paredes S., Villanova L., Brown K., Qiu X., Nabavi N., Mohrin M., Wojnoonski K. (2013). SIRT7 represses Myc activity to suppress ER stress and prevent fatty liver disease. Cell Rep..

[56] Cheng H.L., Mostoslavsky R., Saito S., Manis J.P., Gu Y., Patel P., Bronson R., Appella E., Alt F.W., Chua K.F. (2003). Developmental defects and p53 hyperacetylation in Sir2 homolog (SIRT1)-deficient mice. Proc. Natl. Acad. Sci. USA.

[57] Xu F., Gao Z., Zhang J., Rivera C.A., Yin J., Weng J., Ye J. (2010). Lack of SIRT1 (Mammalian Sirtuin 1) activity leads to liver steatosis in the SIRT1^+/−^ mice: A role of lipid mobilization and inflammation. Endocrinology.

[58] Mayoral R., Osborn O., McNelis J., Johnson A.M., Oh da Y., Izquierdo C.L., Chung H., Li P., Traves P.G., Bandyopadhyay G. (2015). Adipocyte SIRT1 knockout promotes PPAR-γ activity, adipogenesis and insulin sensitivity in chronic-HFD and obesity. Mol. Metab..

[59] Feige J.N., Lagouge M., Canto C., Strehle A., Houten S.M., Milne J.C., Lambert P.D., Mataki C., Elliott P.J., Auwerx J. (2008). Specific SIRT1 activation mimics low energy levels and protects against diet-induced metabolic disorders by enhancing fat oxidation. Cell Metab..

[60] Lagouge M., Argmann C., Gerhart-Hines Z., Meziane H., Lerin C., Daussin F., Messadeq N., Milne J., Lambert P., Elliott P. (2006). Resveratrol improves mitochondrial function and protects against metabolic disease by activating SIRT1 and PGC-1α. Cell.

[61] Sasaki T., Kikuchi O., Shimpuku M., Susanti V.Y., Yokota-Hashimoto H., Taguchi R., Shibusawa N., Sato T., Tang L., Amano K. (2014). Hypothalamic SIRT1 prevents age-associated weight gain by improving leptin sensitivity in mice. Diabetologia.

[62] Lu M., Sarruf D.A., Li P., Osborn O., Sanchez-Alavez M., Talukdar S., Chen A., Bandyopadhyay G., Xu J., Morinaga H. (2013). Neuronal SIRT1 deficiency increases insulin sensitivity in both brain and peripheral tissues. J. Biol. Chem..

[63] Cakir I., Perello M., Lansari O., Messier N.J., Vaslet C.A., Nillni E.A. (2009). Hypothalamic SIRT1 regulates food intake in a rodent model system. PLoS ONE.

[64] Kim M.S., Pak Y.K., Jang P.G., Namkoong C., Choi Y.S., Won J.C., Kim K.S., Kim S.W., Kim H.S., Park J.Y. (2006). Role of hypothalamic Foxo1 in the regulation of food intake and energy homeostasis. Nat. Neurosci..

[65] Toorie A.M., Nillni E.A. (2014). Minireview: Central Sirt1 regulates energy balance via the melanocortin system and alternate pathways. Mol. Endocrinol..

[66] Rothgiesser K.M., Erener S., Waibel S., Lüscher B., Hottiger M.O. (2010). SIRT2 regulates NF-κB dependent gene expression through deacetylation of p65 Lys310. J. Cell Sci..

[67] Nasrin N., Wu X., Fortier E., Feng Y., Bare’ O.C., Chen S., Ren X., Wu Z., Streeper R.S., Bordone L. (2010). SIRT4 regulates fatty acid oxidation and mitochondrial gene expression in liver and muscle cells. J. Biol. Chem..

[68] Yu J., Sadhukhan S., Noriega L.G., Moullan N., He B., Weiss R.S., Lin H., Schoonjans K., Auwerx J. (2013). Metabolic characterization of a SIRT5 deficient mouse model. Sci. Rep..

[69] Ogura M., Nakamura Y., Tanaka D., Zhuang X., Fujita Y., Obara A., Hamasaki A., Hosokawa M., Inagaki N. (2010). Overexpression of SIRT5 confirms its involvement in deacetylation and activation of carbamoyl phosphate synthetase 1. Biochem. Biophys. Res. Commun..

[70] Mostoslavsky R., Chua K.F., Lombard D.B., Pang W.W., Fischer M.R., Gellon L., Liu P., Mostoslavsky G., Franco S., Murphy M.M. (2006). Genomic instability and aging-like phenotype in the absence of mammalian SIRT6. Cell.

[71] Schwer B., Schumacher B., Lombard D.B., Xiao C., Kurtev M.V., Gao J., Schneider J.I., Chai H., Bronson R.T., Tsai L.H. (2010). Neural sirtuin 6 (SIRT6) ablation attenuates somatic growth and causes obesity. Proc. Natl. Acad. Sci. USA.

[72] Kanfi Y., Peshti V., Gil R., Naiman S., Nahum L., Levin E., Kronfeld-Schor N., Cohen H.Y. (2010). SIRT6 protects against pathological damage caused by diet-induced obesity. Aging Cell.

[73] Anderson J.G., Ramadori G., Ioris R.M., Galiè M., Berglund E.D., Coate K.C., Fujikawa T., Pucciarelli S., Moreschini B., Amici A. (2015). Enhanced insulin sensitivity in skeletal muscle and liver by physiological overexpression of SIRT6. Mol. Metab..

[74] Vakhrusheva O., Smolka C., Gajawada P., Kostin S., Boettger T., Kubin T., Braun T., Bober E. (2008). SIRT7 increases stress resistance of cardiomyocytes and prevents apoptosis and inflammatory cardiomyopathy in mice. Circ. Res..

[75] Yoshizawa T., Karim M.F., Sato Y., Senokuchi T., Miyata K., Fukuda T., Go C., Tasaki M., Uchimura K., Kadomatsu T. (2014). SIRT7 controls hepatic lipid metabolism by regulating the ubiquitin-proteasome pathway. Cell Metab..

[76] Crujeiras A.B., Parra D., Goyenechea E., Martínez J.A. (2008). Sirtuin gene expression in human mononuclear cells is modulated by caloric restriction. Eur. J. Clin. Investig..

[77] Clark S.J., Falchi M., Olsson B., Jacobson P., Cauchi S., Balkau B., Marre M., Lantieri O., Andersson J.C., Jernås M. (2012). Association of sirtuin 1 (*SIRT1*) gene SNPs and transcript expression levels with severe obesity. Obesity.

[78] Pedersen S.B., Ølholm J., Paulsen S.K., Bennetzen M.F., Richelsen B. (2008). Low SIRT1 expression, which is upregulated by fasting, in human adipose tissue from obese women. Int. J. Obes..

[79] Dong Y., Guo T., Traurig M., Mason C.C., Kobes S., Perez J., Knowler W.C., Bogardus C., Hanson R.L., Baier L.J. (2011). SIRT1 is associated with a decrease in acute insulin secretion and a sex specific increase in risk for type 2 diabetes in Pima Indians. Mol. Genet. Metab..

[80] Song Y.S., Lee S.K., Jang Y.J., Park H.S., Kim J.H., Lee Y.J., Heo Y.S. (2013). Association between low SIRT1 expression in visceral and subcutaneous adipose tissues and metabolic abnormalities in women with obesity and type 2 diabetes. Diabetes Res. Clin. Pract..

[81] Costa Cdos S., Hammes T.O., Rohden F., Margis R., Bortolotto J.W., Padoin A.V., Mottin C.C., Guaragna R.M. (2010). SIRT1 transcription is decreased in visceral adipose tissue of morbidly obese patients with severe hepatic steatosis. Obes. Surg..

[82] Tarantino G., Finelli C., Scopacasa F., Pasanisi F., Contaldo F., Capone D., Savastano S. (2014). Circulating levels of sirtuin 4, a potential marker of oxidative metabolism, related to coronary artery disease in obese patients suffering from NAFLD, with normal or slightly increased liver enzymes. Oxid. Med. Cell. Longev..

[83] Song R., Xu W., Chen Y., Li Z., Zeng Y., Fu Y. (2011). The expression of Sirtuins 1 and 4 in peripheral blood leukocytes from patients with type 2 diabetes. Eur. J. Histochem..

[84] Bober E., Fang J., Smolka C., Ianni A., Vakhrusheva O., Krüger M., Braun T. (2012). SIRT7 promotes adipogenesis by binding to and inhibiting SIRT1. BMC Proc..

[85] Yamakuchi M., Ferlito M., Lowenstein C.J. (2008). miR-34a repression of SIRT1 regulates apoptosis. Proc. Natl. Acad. Sci. USA.

[86] Zhang S., Zhang D., Yi C., Wang Y., Wang H., Wang J. (2015). MicroRNA-22 functions as a tumor suppressor by targeting SIRT1 in renal cell carcinoma. Oncol. Rep..

[87] Zhou B., Li C., Qi W., Zhang Y., Zhang F., Wu J.X., Hu Y.N., Wu D.M., Liu Y., Yan T.T. (2012). Downregulation of miR-181a upregulates sirtuin-1 (SIRT1) and improves hepatic insulin sensitivity. Diabetologia.

[88] Kim J.K., Noh J.H., Jung K.H., Eun J.W., Bae H.J., Kim M.G., Chang Y.G., Shen Q., Park W.S., Lee J.Y. (2013). Sirtuin7 oncogenic potential in human hepatocellular carcinoma and its regulation by the tumor suppressors MiR-125a-5p and MiR-125b. Hepatology.

[89] Saxena R., Voight B.F., Lyssenko V., Burtt N.P., de Bakker P.I., Chen H., Roix J.J., Kathiresan S., Hirschhorn J.N., Daly M.J. (2007). Genome-wide association analysis identifies loci for type 2 diabetes and triglyceride levels. Science.

[90] Van den Berg S.W., Dollé M.E., Imholz S., van der A D.L., van’t Slot R., Wijmenga C., Verschuren W.M., Strien C., Siezen C.L., Hoebee B. (2009). Genetic variations in regulatory pathways of fatty acid and glucose metabolism are associated with obesity phenotypes: A population-based cohort study. Int. J. Obes..

[91] Peeters A.V., Beckers S., Verrijken A., Mertens I., Roevens P., Peeters P.J., van Hul W., van Gaal L.F. (2008). Association of *SIRT1* gene variation with visceral obesity. Hum. Genet..

[92] Zillikens M.C., van Meurs J.B., Rivadeneira F., Amin N., Hofman A., Oostra B.A., Sijbrands E.J., Witteman J.C., Pols H.A., van Duijn C.M. (2009). *SIRT1* genetic variation is related to BMI and risk of obesity. Diabetes.

[93] Weyrich P., Machicao F., Reinhardt J., Machann J., Schick F., Tschritter O., Stefan N., Fritsche A., Häring H.U. (2008). *SIRT1* genetic variants associate with the metabolic response of Caucasians to a controlled lifestyle intervention-the TULIP Study. BMC Med. Genet..

[94] Shimoyama Y., Suzuki K., Hamajima N., Niwa T. (2011). *SIRT1* gene polymorphisms are associated with body fat and blood pressure in Japanese. Transl. Res..

[95] Kilic U., Gok O., Elibol-Can B., Ozgen I.T., Erenberk U., Uysal O., Dundaroz M.R. (2015). *SIRT1* gene variants are related to risk of childhood obesity. Eur. J. Pediatr..

[96] Figarska S.M., Vonk J.M., Boezen H.M. (2013). SIRT1 polymorphism, long-term survival and glucose tolerance in the general population. PLoS ONE.

[97] Zarrabeitia M.T., Valero C., Martín-Escudero J.C., Olmos J.M., Bolado-Carrancio A., de Sande-Nacarino E.L., Rodríguez-Rey J.C., Sainz J., Riancho J.A. (2012). Association study of sirtuin 1 polymorphisms with bone mineral density and body mass index. Arch. Med. Res..

[98] Kedenko L., Lamina C., Kedenko I., Kollerits B., Kiesslich T., Iglseder B., Kronenberg F., Paulweber B. (2014). Genetic polymorphisms at SIRT1 and FOXO1 are associated with carotid atherosclerosis in the SAPHIR cohort. BMC Med. Genet..

[99] Dong C., Della-Morte D., Wang L., Cabral D., Beecham A., McClendon M.S., Luca C.C., Blanton S.H., Sacco R.L., Rundek T. (2011). Association of the sirtuin and mitochondrial uncoupling protein genes with carotid plaque. PLoS ONE.

[100] Della-Morte D., Dong C., Bartels S., Cabral D., Blanton S.H., Sacco R.L., Rundek T. (2012). Association of the sirtuin and mitochondrial uncoupling protein genes with carotid intima-media thickness. Transl. Res..

[101] Dong C., Della-Morte D., Cabral D., Wang L., Blanton S.H., Seemant C., Sacco R.L., Rundek T. (2015). Sirtuin/uncoupling protein gene variants and carotid plaque area and morphology. Int. J. Stroke.

[102] Cui Y., Wang H., Chen H., Pang S., Wang L., Liu D., Yan B. (2012). Genetic analysis of the *SIRT1* gene promoter in myocardial infarction. Biochem. Biophys. Res. Commun..

[103] Kilic U., Gok O., Bacaksiz A., Izmirli M., Elibol-Can B., Uysal O. (2014). *SIRT1* gene polymorphisms affect the protein expression in cardiovascular diseases. PLoS ONE.

[104] Cruz M., Valladares-Salgado A., Garcia-Mena J., Ross K., Edwards M., Angeles-Martinez J., Ortega-Camarillo C., de la Peña J.E., Burguete-Garcia A.I., Wacher-Rodarte N. (2010). Candidate gene association study conditioning on individual ancestry in patients with type 2 diabetes and metabolic syndrome from Mexico city. Diabetes Metab. Res. Rev..

[105] Maeda S., Koya D., Araki S., Babazono T., Umezono T., Toyoda M., Kawai K., Imanishi M., Uzu T., Suzuki D. (2011). Association between single nucleotide polymorphisms within genes encoding sirtuin families and diabetic nephropathy in Japanese subjects with type 2 diabetes. Clin. Exp. Nephrol..

[106] Shimoyama Y., Mitsuda Y., Tsuruta Y., Suzuki K., Hamajima N., Niwa T. (2012). *Sirtuin 1* gene polymorphisms are associated with cholesterol metabolism and coronary artery calcification in Japanese hemodialysis patients. J. Ren. Nutr..

[107] Elghobashy Y., Tayel S., ALrefai A., Khamis S., Elbarbary H. (2014). Relation of *sirtuin 1* gene polymorphism with lipid profile in hemodialysis patients. Br. Biotechnol. J..

[108] Mellini P., Valente S., Mai A. (2015). Sirtuin modulators: An updated patent review (2012–2014). Expert Opin. Ther. Pat..

[109] Howitz K.T., Bitterman K.J., Cohen H.Y., Lamming D.W., Lavu S., Wood J.G., Zipkin R.E., Chung P., Kisielewski A., Zhang L.L. (2003). Small molecule activators of sirtuins extend *Saccharomyces cerevisiae* lifespan. Nature.

[110] Rayalam S., Yang J.Y., Ambati S., Della-Fera M.A., Baile C.A. (2008). Resveratrol induces apoptosis and inhibits adipogenesis in 3T3-L1 adipocytes. Phytother. Res..

[111] Aguirre L., Fernández-Quintela A., Arias N., Portillo M.P. (2014). Resveratrol: Anti-obesity mechanisms of action. Molecules.

[112] Pearson K.J., Baur J.A., Lewis K.N., Peshkin L., Price N.L., Labinskyy N., Swindell W.R., Kamara D., Minor R.K., Perez E. (2008). Resveratrol delays age-related deterioration and mimics transcriptional aspects of dietary restriction without extending life span. Cell Metab..

[113] Baur J.A., Pearson K.J., Price N.L., Jamieson H.A., Lerin C., Kalra A., Prabhu V.V., Allard J.S., Lopez-Lluch G., Lewis K. (2006). Resveratrol improves health and survival of mice on a high-calorie diet. Nature.

[114] Kim S., Jin Y., Choi Y., Park T. (2011). Resveratrol exerts anti-obesity effects via mechanisms involving down-regulation of adipogenic and inflammatory processes in mice. Biochem. Pharmacol..

[115] De Ligt M., Timmers S., Schrauwen P. (2015). Resveratrol and obesity: Can resveratrol relieve metabolic disturbances?. Biochim. Biophys. Acta.

[116] Timmers S., Konings E., Bilet L., Houtkooper R.H., van de Weijer T., Goossens G.H., Hoeks J., van der Krieken S., Ryu D., Kersten S. (2011). Calorie restriction-like effects of 30 days of resveratrol supplementation on energy metabolism and metabolic profile in obese humans. Cell Metab..

[117] Konings E., Timmers S., Boekschoten M.V., Goossens G.H., Jocken J.W., Afman L.A., Müller M., Schrauwen P., Mariman E.C., Blaak E.E. (2014). The effects of 30 days resveratrol supplementation on adipose tissue morphology and gene expression patterns in obese men. Int. J. Obes..

[118] Smith J.J., Kenney R.D., Gagne D.J., Frushour B.P., Ladd W., Galonek H.L., Israelian K., Song J., Razvadauskaite G., Lynch A.V. (2009). Small molecule activators of SIRT1 replicate signaling pathways triggered by calorie restriction *in vivo*. BMC Syst. Biol..

[119] Howells L.M., Berry D.P., Elliott P.J., Jacobson E.W., Hoffmann E., Hegarty B., Brown K., Steward W.P., Gescher A.J. (2011). Phase I randomized, double-blind pilot study of micronized resveratrol (SRT501) in patients with hepatic metastases—Safety, pharmacokinetics, and pharmacodynamics. Cancer Prev. Res..

[120] Goh K.P., Lee H.Y., Lau D.P., Supaat W., Chan Y.H., Koh A.F. (2014). Effects of resveratrol in patients with type 2 diabetes mellitus on skeletal muscle SIRT1 expression and energy expenditure. Int. J. Sport Nutr. Exerc. Metab..

[121] Faghihzadeh F., Adibi P., Hekmatdoost A. (2015). The effects of resveratrol supplementation on cardiovascular risk factors in patients with non-alcoholic fatty liver disease: A randomised, double-blind, placebo-controlled study. Br. J. Nutr..

[122] Poulsen M.M., Vestergaard P.F., Clasen B.F., Radko Y., Christensen L.P., Stødkilde-Jørgensen H., Møller N., Jessen N., Pedersen S.B., Jørgensen J.O. (2013). High-dose resveratrol supplementation in obese men: An investigator-initiated, randomized, placebo-controlled clinical trial of substrate metabolism, insulin sensitivity, and body composition. Diabetes.

[123] Yoshino J., Conte C., Fontana L., Mittendorfer B., Imai S., Schechtman K.B., Gu C., Kunz I., Rossi Fanelli F., Patterson B.W. (2012). Resveratrol supplementation does not improve metabolic function in nonobese women with normal glucose tolerance. Cell Metab..

[124] Baksi A., Kraydashenko O., Zalevkaya A., Stets R., Elliott P., Haddad J., Hoffmann E., Vlasuk G.P., Jacobson E.W. (2014). A phase II, randomized, placebo-controlled, double-blind, multi-dose study of SRT2104, a SIRT1 activator, in subjects with type 2 diabetes. Br. J. Clin. Pharmacol..

[125] Milne J.C., Lambert P.D., Schenk S., Carney D.P., Smith J.J., Gagne D.J., Jin L., Boss O., Perni R.B., Vu C.B. (2007). Small molecule activators of SIRT1 as therapeutics for the treatment of type 2 diabetes. Nature.

[126] Yamazaki Y., Usui I., Kanatani Y., Matsuya Y., Tsuneyama K., Fujisaka S., Bukhari A., Suzuki H., Senda S., Imanishi S. (2009). Treatment with SRT1720, a SIRT1 activator, ameliorates fatty liver with reduced expression of lipogenic enzymes in MSG mice. Am. J. Physiol. Endocrinol. Metab..

[127] Pacholec M., Bleasdale J.E., Chrunyk B., Cunningham D., Flynn D., Garofalo R.S., Griffith D., Griffor M., Loulakis P., Pabst B. (2010). SRT1720, SRT2183, SRT1460, and resveratrol are not direct activators of SIRT1. J. Biol. Chem..

[128] Cantó C., Gerhart-Hines Z., Feige J.N., Lagouge M., Noriega L., Milne J.C., Elliott P.J., Puigserver P., Auwerx J. (2009). AMPK regulates energy expenditure by modulating NAD^+^ metabolism and SIRT1 activity. Nature.

[129] Villalba J.M., Alcaín F.J. (2012). Sirtuin activators and inhibitors. Biofactors.

